# Synthesis of Up-Conversion CaTiO_3_: Er^3+^ Films on Titanium by Anodization and Hydrothermal Method for Biomedical Applications

**DOI:** 10.3390/ma17133376

**Published:** 2024-07-08

**Authors:** Nguyen Thi Thanh Tuyen, Ta Quoc Tuan, Le Van Toan, Le Thi Tam, Vuong-Hung Pham

**Affiliations:** 1 School of Materials Science and Engineering, Hanoi University of Science and Technology (HUST), No. 01 Dai Co Viet, Hanoi 100000, Vietnam; nguyenthanhtuyenvatly37@gmail.com (N.T.T.T.); tuan.taquoc@hust.edu.vn (T.Q.T.); levantoan2011@gmail.com (L.V.T.); tam.lethi@hust.edu.vn (L.T.T.); 2 Laboratory of Biomedical Materials, Hanoi University of Science and Technology (HUST), No. 01 Dai Co Viet, Hanoi 100000, Vietnam; 3 Department of Physics and Chemical Engineering, Le Quy Don Technical University, 236 Hoang Quoc Viet Road, Hanoi 11917, Vietnam

**Keywords:** CaTiO_3_: Er^3+^ phosphor, hydrophilicity, up-conversion emission, hydrothermal method

## Abstract

The present study investigates the effects of Er^3+^ doping content on the microstructure and up-conversion emission properties of CaTiO_3_: Er^3+^ phosphors as a potential material in biomedical applications. The CaTiO_3_: *x*%Er^3+^ (*x* = 0.5, 1.0, 1.5, 2.0, 2.5, and 3.0%) films were synthesized on Ti substrates by a hydrothermal reaction at 200 °C for 24 h. The SEM image showed the formation of cubic nanorod CaTiO_3_: Er^3+^ films with a mean edge size value of (1–5) μm. When excited with 980 nm light, the CaTiO_3_: Er^3+^ films emitted a strong green band and a weak red band of Er^3+^ ions located at 543, 661, and 740 nm. The CaTiO_3_: Er^3+^ film exhibited excellent surface hydrophilicity with a contact angle of ~zero and good biocompatibility against baby hamster kidney (BHK) cells. CaTiO_3_: Er^3+^ films emerge as promising materials for different applications in the biomedical field.

## 1. Introduction

CaTiO_3_ emerges as a promising host material for luminescence-based applications and biomedicine due to its exceptional combination of properties: high chemical durability, long luminescence lifetime, high color rendering index, low power consumption, and biocompatibility. Previous work also reported the biocompatibility of CaTiO_3_ particles [1]. However, to our knowledge, the UC luminescence and cell compatibility of CaTiO_3_: Er^3+^ films on Ti substrate synthesized by a hydrothermal method have yet to be well documented.

Semiconductors doped with rare earth (RE) ions are potential materials for a variety of applications, such as antimicrobial activities, photocatalytic treatment, and environmental purification [2,3]. Perovskite and oxide-based materials are effective hosts for the up-luminescence of RE ions [4,5] because of their low phonon energy, low cost, and easy synthesis [6,7]. Recent research of luminescent RE ions has focused on the Er^3+^ ion thanks to its unique electronic and optical properties and various applications, such as three-dimensional displays [8], solar cells [9,10], temperature sensors [11], photocatalysts [12,13], and biomedical field applications [14,15]. The UC luminescence of CaTiO_3_: Er^3+^ particles is highly attractive for various applications, including drug delivery, tumor therapy, and cell imaging. Er^3+^ ions can be substitutionally doped into the CaTiO_3_ lattice, where they occupy well-defined positions because the material’s crystal structure and the close correspondence between the ionic radii of Er^3+^ and Ca^2+^ ions contribute to the prevention of lanthanide element release [16,17]. The characteristics of Er^3+^ emissions from ^4^F_9/2_→^4^I_15/2_ (red) and ^4^S_3/2_, ^2^H_11/2_→^4^I_15/2_ (green) of the material displayed various transitions under room temperature conditions. The UC luminescence properties of the material can be tailored by adjusting the Er^3+^ doping concentration and the UC photon excitation mechanism [1,18,19]. 

The hydrophilic property plays a crucial role in numerous biomedical applications. For instance, it serves several purposes, including tissue integration, enhanced cell interactions, and drug delivery. In the context of tissue integration, hydrophilic materials possess the ability to absorb water-based substances from the surrounding environment, enabling them to integrate better with body tissues. This can potentially reduce the risk of implant rejection and improve the long-term functionality of implants [20]. Additionally, the hydrophilic property can foster a favorable environment for cell adhesion and growth, promoting wound healing and tissue regeneration around the implant [21]. Furthermore, hydrophilic materials can be utilized to deliver drugs or growth factors to specific locations within the body, enhancing treatment efficacy and minimizing side effects [22].

In this study, we reported the hydrothermal synthesis of up-conversion (UC) emission of CaTiO_3_: Er^3+^ film on Ti and discussed the influence of Er^3+^ concentrations on the UC luminescence of CaTiO_3_: Er^3+^ film. We also investigated the hydrophilic properties and cell compatibility of the CaTiO_3_: Er^3+^ film for biomedical applications. 

## 2. Experimental

### 2.1. Synthesis of CaTiO_3_: x%Er^3+^

CaTiO_3_: Er^3+^ films were synthesized using the hydrothermal method. Calcium hydroxide [Ca(OH)_2,_ Merck (Rahway, NJ, USA), 99.9%], sodium hydroxide (NaOH, Merck, ≥97.0%), TiO_2_ nanotubes template, and erbium chloride hexahydrate (ErCl_3_·6H_2_O, Merck, 99.9%) were used as starting materials. TiO_2_ nanotubes templates were synthesized using the anodization method as reported in previous work [18]. A total of 7.4 mg of Calcium hydroxide (Ca(OH)_2_) was dissolved in 40 mL of H_2_O, and 3.2 mg of NaOH was dissolved in 10 mL of H_2_O. After stirring the two mixtures for 15 min at room temperature, the mixtures were combined under continued magnetic stirring. Next, 7.8 mg of ErCl_3_·6H_2_O was dissolved in H_2_O and slowly added to the beaker with the previous solution. Finally, the mixture solution was used for the hydrothermal synthesis of CaTiO_3_ at 200 °C for 24 h. The synthesized CaTiO_3_: Er^3+^ powder was subjected to a high-temperature treatment (annealing) at 800 °C for 2 h to obtain the final samples.

### 2.2. Characterization

#### 2.2.1. Physicochemical Analysis Methods

The crystallographic properties of the obtained samples were investigated using X-ray techniques powder diffractometer (XRD, Siemens D500, Munich, Germany). Field emission scanning electron microscopy (FESEM, FE-SEM, JEOL JSM-7600F) was employed to investigate the morphology of CaTiO_3_: Er^3+^. **FESEM equipped with EDS (Gatan, UK) was used for the elemental analysis of the samples.** A special instrument (NANO LOG spectrophotometer) was used to measure the light emission properties (luminescence) of CaTiO_3_: Er^3+^. The instrument used two light sources: a high-power xenon lamp and a specific wavelength laser 980 nm. All the analyses were performed at room temperature. Water contact angle measurements were performed to assess the surface properties of the films by using a digital camera at room temperature. Close-up pictures were taken of tiny drops (about 1 microliter) of water placed on the top surface of each film.

#### 2.2.2. Biocompatibility Assessment Methods

To assess cell compatibility in vitro, both the titanium substrate and CaTiO_3_: Er^3+^ films were sterilized through autoclaving. This sterilization process involved exposure to high pressure steam at 121 °C for 60 min. Meanwhile, baby hamster kidney cells (BHK cells) were cultured in a growth medium called DMEM at 27 °C. The culture environment was maintained with humidified air and 5% CO_2_. Cells at the same concentration and density were then applied to both the titanium and CaTiO_3_: Er^3+^ films. A special microscope with lasers (confocal laser scanning microscopy, FV3000RS, Olympus, Tokyo, Japan) was used to see how well the cells attached to the surfaces. After culturing for 48 h, the BHK cells on the CaTiO_3_: Er^3+^ films and the Ti substrates underwent fixation (4% paraformaldehyde/PBS, 10 min), washing, permeabilization (0.1% Triton X-100/PBS, 5 min), washing, and fluorescent phalloidin labeling (45 min). Cell nuclei were specifically stained with a fluorescent dye called DAPI for a duration of 5 min. The stained cells adhering to the samples were mounted onto glass cover slips for subsequent observation of cell attachment. Cell growth was evaluated by CellTiter 96^®^ AQ_ueous_ one solution compound, which contains a tetrazolium compound [3-(4,5-dimethylthiazol-2-yl)-5-(3-carboxymethoxyphenyl)-2-(4-sulfophenyl)-2H-tetrazolium, inner salt; MTS]. The quantity of the formazan product, which is measured by the absorbance at 490 nm using a microreader (Chromate 4300 Microplate Reader, Awareness Technology, Palm City, FL, USA), is directly proportional to the number of living cells on the sample.

## 3. Results and Discussion

### 3.1. Results of Material Synthesis

**Figure 1:**
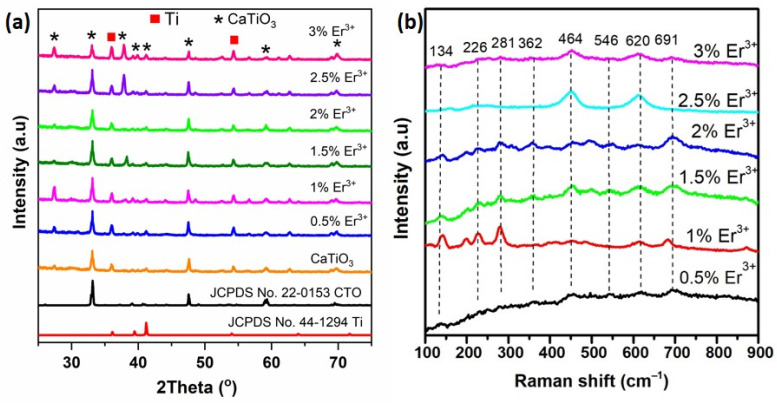
(**a**) XRD patterns of CaTiO_3_: *x*Er^3+^ films (*x* = 0.5, 1.0, 1.5, 2.0, 2.5, and 3.0 mol%); (**b**) Raman spectra of CaTiO_3_: Er^3+^ films.

Following the fabrication of the material with varying Er dopant concentrations, the morphological and structural characterization of these samples was analyzed. The successful fabrication of CaTiO_3_: Er^3+^ thin films was clearly demonstrated by using analytical techniques, including XRD, Raman, EDS, and SEM. Figure 1a shows the XRD patterns of the as-synthesized samples with different Er^3+^ doping content. Based on JCPDS No. 22–0153 reference database, all diffraction patterns of the samples correspond to pure orthorhombic phase CaTiO_3_ with space group *Pbnm*. The orthorhombic structure of CaTiO_3_ contains a Ca^2+^ ion with eight coordinates (CaO_8_) and the Ti^4+^ ion with six coordinates in an octahedron (TiO_6_) [5]. Ca^2+^ atoms at the dodecahedron (CaO_8_) site are easily replaced by Er^3+^, while Ti^4+^ atoms continue to be unaltered at the TiO_6_ site. Given the similarity of the ionic radii of Er^3+^ (1.19 Å, coordination number = 12) and Ca^2+^ (1.34 Å, coordination number = 12), RE^3+^ can replace Ca^2+^ in the CaTiO_3_ structure [1]. To compensate for charge imbalances caused by lattice defects, a substitution process occurs, including Ca^2+^ (V’_Ca_) and/or O^2−^ vacancies (V_O_•) [19,23]. The diffraction profile of the *Pbnm* space exhibits typical (hkl) planes such as (111), (121), (031), (220), (040), (042), and (242) at 27.4°, 33.1°, 39.4°, 41.2°, 47.03°, 59.1°, and 69.8°, respectively. The most dominant diffraction peaks appeared and were centered at 2*θ* = 33.1°, corresponding to the (121) plane of the CaTiO_3_ phase. As shown in Figure 1a, The XRD model of the CaTiO_3_ film with optimized Er^3+^ concentration suggests the presence of a minor phase (peaks marked by symbol ▪) corresponding to Ti (JCPDS No. 44–1294), but this phenomenon did not affect much the phase of CaTiO_3_ and optical properties. These results indicated the successful synthesis of the crystalline structure of CaTiO_3_ films. Moreover, the XRD result revealed that the intensity of CaTiO_3_: Er^3+^ diffraction peaks increases gradually with an increasing dopant concentration. When the concentration of Er^3+^ is increased to 2.5%, two characteristic peaks, 33.1° and 47.03°, emerge with relatively high intensity and sharpness. This indicates that the crystal structure of CaTiO_3_: Er^3+^ is well formed.

Raman spectroscopy serves as a valuable tool for studying symmetry changes in various compounds. In the case of the CaTiO_3_ material, twenty-four Raman-active modes were identified within its orthorhombic *Pbnm* crystal structure (with Z^B^ = 4), featuring four molecular units within the primitive cell. The material’s irreducible representation is denoted as *Γ*_Raman,_*_Pbnm_* = 7*A*_g_ + 5*B*_1g_ + 7*B*_2g_ + 5*B*_3g_. Figure 1b depicts the Raman spectra of CaTiO_3_: Er^3+^ films span the frequency scope of 100–900 cm^−1^. Raman-active modes at 134 cm^−1^ is attributed to the oscillation of Ca bonded to the TiO_3_ (Ca–TiO_3_) lattice. Modes at 226, 244, 281, and 362 cm^−1^ are linked to O–Ti–O bending modes, while those at 464 and 495 cm^−1^ correspond to Ti–O_6_ twisted modes (bending or internal oscillation of the oxygen cage), with a second large band observed in the scope of 600–700 cm^−1^. These observations agree with previous works [24,25,26,27]. 

**Figure 2:**
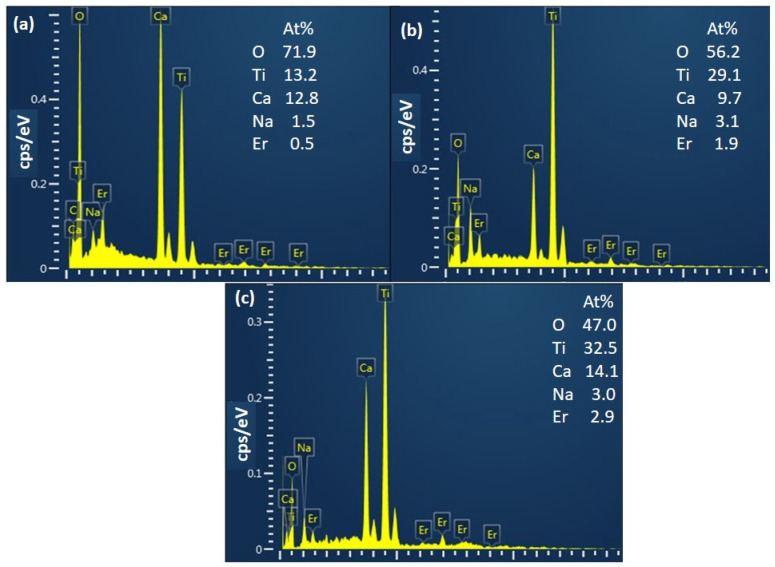
EDS spectra of (**a**) CaTiO_3_: 0.5%Er^3+^, (**b**) CaTiO_3_: 2%Er^3+^, and (**c**) CaTiO_3_: 3%Er^3+^ samples.

Figure 2 shows the EDS spectra of the CaTiO_3_: *x*Er^3+^ films (*x* = 0.5, 2.0, and 3.0 mol%). All samples show attendance of O, Ca, Ti, Er, and Na elements. The presence of Na could be due to the residual input solution. Analysis revealed no traces of other impurities, suggesting the high purity of the synthesized phosphors.

**Figure 3:**
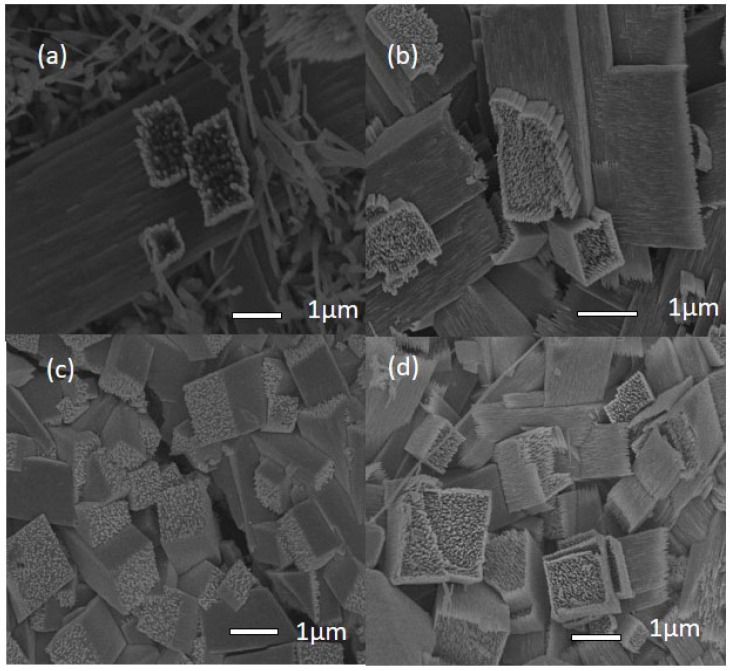
FESEM images of the CaTiO_3_: Er^3+^ films with divergent Er^3+^ concentration; (**a**) 0.5% Er^3+^ films; (**b**) 1%Er^3+^; (**c**) 1.5% Er^3+^; and (**d**) 2%Er^3+^.

Figure 3 shows the FESEM picture of the CaTiO_3_: Er^3+^ films. As shown in Figure 3, when the CaTiO_3_ is doped at a concentration of 0.5% Er^3+^, the material begins to form fiber clumps. Increasing the doping concentration of (1–2)% Er^3+^ leads to the formation of increasingly homogeneous square-shaped fiber bundles. The CaTiO_3_: Er^3+^ films consist of uniform cubic particles with a bar length of ~(1–5) μm and a width of ~(1–2) μm. The results of this SEM analysis are consistent with the previously analyzed XRD data.

### 3.2. Material Properties Evaluation Findings

#### 3.2.1. UC Luminescence Properties

**Figure 4:**
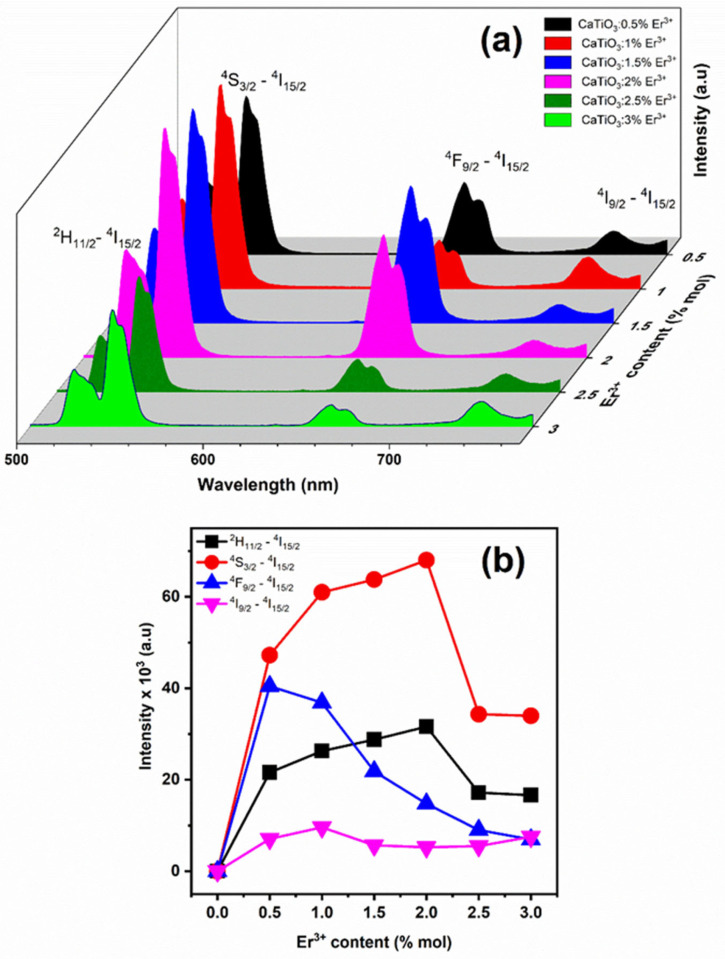
(**a**) UC emission spectra of CaTiO_3_:x% Er^3+^ films (x = 0.5, 1.0, 1.5, 2.0, 2.5, and 3.0 mol%). (**b**) The intensity of green (^2^H_11/2_, ^4^S_3/2_–^4^I_15/2_) and red (^4^F_9/2_–^4^I_15/2_) in UC emission as a function of Er^3+^ doping concentrations under excitation at 980 nm.

Following the successful fabrication of the material, we proceeded to investigate its optical properties when doped with rare earth erbium (Er^3+^) ions. Samples doped with Er^3+^ at varying concentrations, ranging from 0.5% to 3%, were employed to investigate their optical properties. Figure 4a,b depict the UC luminescence spectra for all samples with an excitation wavelength of 980 nm. Upon 980 nm excitation, all samples show intense green UC emission bands at 523/550 nm (^2^H_11/2_→^4^I_15/2_/^4^S_3/2_→^4^I_15/2_), a faint red emission band at 650/680 nm (^4^F_9/2_→^4^I_15/2_), and a far-red band at 740 nm (^4^I_9/2_→^4^I_15/2_). Across all samples with different Er^3+^ doping concentrations, a robust green emission band and a diminishing red emission band are consistently observed. The dominant emissions are located in the green luminescence range for CaTiO_3_: Er^3+^ films. The comparative intensity of the three energy level transitions ^4^F_9/2_→^4^I_15/2_, ^2^H_11/2_→^4^I_15/2_, and ^4^S_3/2_→^4^I_15/2_ increases with increasing Er^3+^ concentration. The peak up-conversion (UC) emission intensity for the CaTiO_3_: Er^3+^ films was identified by upholding the optimal convergence of the Er^3+^ activator. The discernible red and blue emission peaks suggest enhanced conversion of infrared light to visible light, attributed to the synergistic effect of Er^3+^ ion co-doping in CaTiO_3_. This phenomenon notably broadens the emission spectrum in the visible region, expanding the range of electromagnetic waves emitted [28,29]. As illustrated in Figure 4a, the luminescence intensity of the material varies with the doping concentration. When the Er^3+^ dopant concentration is increased from 0.5% to 2%, the luminescence intensity also increases. However, further increasing the dopant concentration leads to a decrease in luminescence intensity. This phenomenon of intensity reduction is known as luminescence quenching.

**Figure 5:**
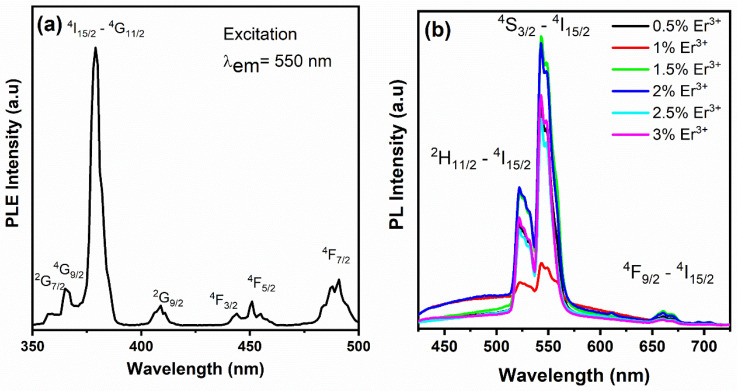
(**a**) PLE excitation spectra of CaTiO_3_: 2% Er^3+^ films under emission at 550 nm. (**b**) PL emission spectra of CaTiO_3_: x% Er^3+^ (x = 0.5, 1.0, 1.5, 2.0, 2.5 and 3.0 mol%), under excitation at 980 nm.

The excitation spectrum of the 550 nm emission band (green) in 2.0 mol% Er^3+^–doped CaTiO_3_ films is depicted in Figure 5a. The seven bands with centers at 357, 365, 379, 408, 445, 451, and 491 nm correspond to the excited levels, namely, ^2^G_7/2_, ^4^G_9/2_, ^4^G_11/2_, ^2^G_9/2_, ^4^F_3/2_, ^4^F_5/2_, and ^4^F_7/2_, respectively. The most prominent peak at 379 nm aligns with the ^4^I_15/2_→^4^G_11/2_ transition. The excitation spectrum reveals various pathways to obtain green luminescence from the CaTiO_3_ films. CaTiO_3_: Er^3+^ emits bright green emission when the films are excited by 390 nm laser light. The visible emission spectra for samples doped with 0.5, 1.0, 1.5, 2.0, 2.5, and 3.0 mol% Er^3+^ are presented in Figure 5b. The two prominent green bands in the spectral ranges of 522–535 and 537–560 nm align with the ^2^H_11/2_→^4^I_15/2_ and ^4^S_3/2_→^4^I_15/2_ transitions, respectively. The fainter red band in the 650–675 nm region aligns with the ^4^F_9/2_→^4^I_15/2_ transition [30].

**Figure 6:**
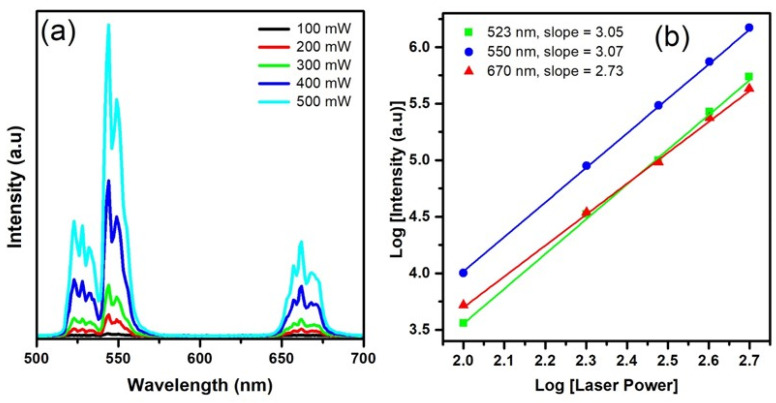
(**a**) UC emission spectra of the 2% Er^3+^ sample with different power density. (**b**) The log–log plots of red and green emission intensity as a function of the excitation laser power.

**Figure 7:**
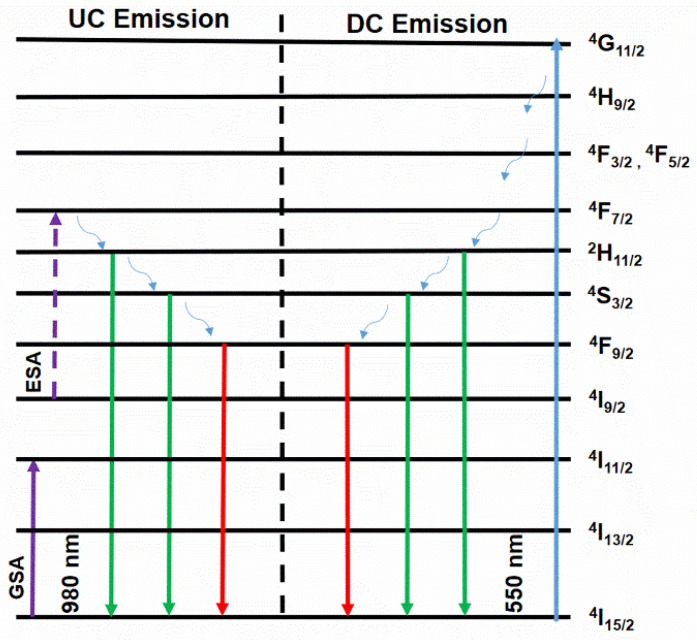
Energy level diagram of CaTiO_3_: Er^3+^ under excitation at 980 nm.

The rapport between up-conversion intensity (I_upc_) bands and pump power excitation (P_pump_) was examined to elucidate the up-conversion process in Er^3+^ co-doped films, as illustrated in Figure 6a,b. Understanding the power accessorial of up-conversion luminescence is crucial for unraveling the up-conversion convert mechanism. Typically, when up-conversion luminescence is generated at low pump intensity, the correlation between up-conversion emission intensity (I) and pump power (P) can be written as I_upc_ ∝ (P_pump_)^n^ or log (I_upc_) ∝ n log (P_pump_), where ‘n’ signifies the number of photons required to be excited to attain the up-conversion emitting level [31]. Figure 6a shows the power-dependent UC fluorescence spectra, and Figure 6b shows that the integrated intensity of the green and red emissions of CaTiO_3_: Er^3+^ (2%) varies with pump power on the double logarithmic scale. Figure 6b shows that the log–log power accessorial slopes of 523, 550, and 670 nm are 3.05, 3.07, and 2.73, respectively. Our findings suggest that the green and red up-conversion (UC) luminescence of Er^3+^ stems from three-photon processes in the circumstances of the CaTiO_3_: Er^3+^. Supported by the observed quadratic dependence on pump power and the alignment of energy levels, the most likely transitions responsible for the UC emissions are illustrated in Figure 7.

#### 3.2.2. UC Mechanism of Er-Doped Systems

Er (^4^I_15/2_) + hν_980nm_→Er (^4^I_11/2_) (1)

Er (^2^h_11/2_)→Er (^4^I_15/2_) + hν_523nm_ (2)

Er (^4^I_11/2_) + Er (^4^I_11/2_)→Er (^4^S_3/2_) + Er (^2^I_15/2_)→Er (^4^I_15/2_) + hν_550nm_ (3)

Er (^4^I_11/2_) + Er (^4^I_13/2_)→Er (^4^F_9/2_) + Er (^2^I_15/2_)→Er (^4^I_15/2_) + hν_675nm_ (4)

Figure 7 shows the system’s energy level diagram to understand the phosphor’s dual-mode green emission behavior. Several steps are proposed for selectively enhancing green UC emission of CaTiO_3_: Er^3+^ phosphors [9,32]. 

Step 1: The process commences with the absorption of a photon by the electron at the ^4^I_15/2_ state, elevating it to the ^4^I_11/2_ level. This initial step, known as ground state absorption (GSA), is pivotal to the system’s behavior.

Step 2: The electron at the ^4^I_11/2_ level absorbs a second photon and transitions to a higher energy excited state, the ^2^F_7/2_ level, by excited condition absorption (ESA). It is notable owing to the unstable 2F_7/2_ state that leads to no radiative relaxation of the electron to the ^2^H_11/2_ and ^4^S_3/2_ levels (two meta-stable levels).

Step 3: The electrons in ^2^H_11/2_ and ^4^S_3/2_ return to the ground condition ^4^I_15/2_, producing intense green emission. Meanwhile, a few electrons underwent non-radiative relaxation from the ^4^S_3/2_ level to the ^4^F_9/2_ level and then moved to the initial condition (^4^I_15/2_), forming a weak red UC emission.

The Er^3+^ ions initially undergo excitation from the ^2^F_7/2_ to ^2^F_5/2_ level by absorbing laser photons (ground/excited state absorption, *GSA/ESA*) due to the strong absorption at wavelength 980 nm. Under 980 nm excitation, the ^4^I_11/2_ level of Er^3+^ becomes populated by absorbing a single infrared photon from the ^4^I_15/2_ level (called ground state absorption, GSA), as depicted in Equation (1). Through the nonradiative relaxation (NR) progress from the ^4^F_7/2_ level to the ^2^H_11/2_/^4^S_3/2_ (two meta-stable levels) lower energy level, practically all of the electrons in the ^2^H_11/2_/^4^S_3/2_ level progressed down to the first state ^4^I_15/2_ of Er^3+^, which form intense green emissions (523–550 nm). The electrons in the Er^3+^ (^4^F_7/2_) excited state could decay to the ^2^H_11/2_ excited state through a process called thermal radiation. This process emits a 523 nm photon as the electrons return to their ground state, Er^3+^ (^4^I_15/2_), as illustrated in Equation (2). Moreover, a part of electrons in the Er^3+^ (^4^I_11/2_) stimulated state can transfer energy to an adjacent electron within a similar energy level. Consequently, an electron transition from Er^3+^ (^4^I_11/2_) to Er^3+^ (^4^S_3/2_) stimulated state ensues, triggering radiative relaxation to Er^3+^ (^4^I_15/2_) along with the emission of a 550 nm green photon, as described by Equation (3). The electrons in the ^4^S_3/2_ level undergo no radiative relaxation to the ^4^F_9/2_ level; the next step is their return to the initial state of ^4^I_15/2_, creating a red emission band, as indicated in Equation (4). The energy of the ^4^S_3/2_ level surpasses that of the ^4^F_9/2_ level, resulting in a subdued red emission band.

In the direct current (DC) emission, the strong green emission and weak red emission can be explained as follows: Initially, Er^3+^ ions in the basic state undergo excitation to the ^4^G_11/2_ level (refer to Figure 7) through a xenon lamp with excitation wavelengths of 379 nm. Subsequently, a majority of the electrons in the ^4^G_11/2_ level relax to the ^4^S_3/2_ no radiative (NR) level with the swiftest multi-phonon relaxation rate, followed by their return to the radiative basic state, ^4^I_15/2_, leading to the generation of two green emission bands. Notably, there is minimal excitation of electrons to the ^4^I_11/2_ state, leading to a limited electron population in the ^4^F_9/2_ level. Therefore, red emission occurs with weak intensity. Our findings indicate that the proposed mechanism effectively explains the dual-mode green emission observed in the CaTiO_3_: Er^3+^ films from the perspective of the electronic structure scale.

### 3.3. Biocompatibility of CaTiO_3_: Er^3+^

**Figure 8:**
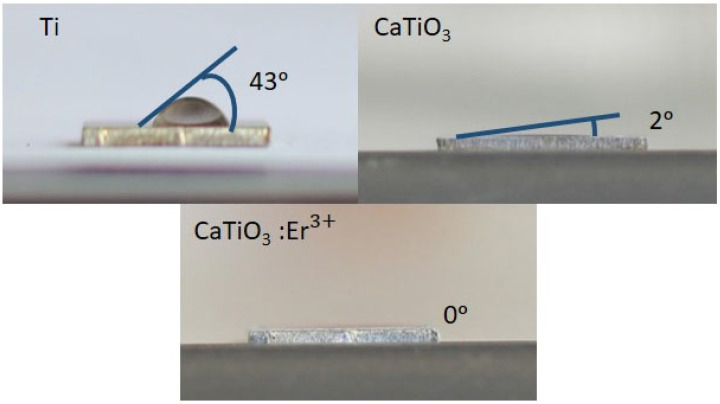
Contact angles of Ti substrate, CaTiO_3_, and CaTiO_3_: Er^3+^ films.

To confirm the biocompatibility of the material, the research team prepared a CaTiO_3_: 2%Er^3+^ sample and conducted a study on its hydrophilicity. The hydrophilicity of a material is determined by using a contact angle measurement technique. In this work, we used the contact angle method to verify the hydrophilic properties of the Ti substrate, CaTiO_3_ film, and CaTiO_3_: Er^3+^ films. Interestingly, as shown in Figure 8, the result of all CaTiO_3_: Er^3+^ films were considerably lower than that of the Ti substrate and CaTiO_3_ films. Among the three kinds of films, the CaTiO_3_: Er^3+^ films had the most evident decrease in contact angle, which indicated the best improvement in hydrophilic. Hence, surface transformation from the hydrophobic Ti to hydrophilic CaTiO_3_: Er^3+^ films can be used to design water dispersible film phosphors in bio-medical fields. The findings demonstrate that the fabricated material possesses promising biomedical applications, particularly in the realm of implants. The material’s hydrophilic plays a crucial role in enhancing cell adhesion and growth, which can potentially promote wound healing and tissue regeneration in the surrounding area. Furthermore, highly hydrophilic materials can be utilized for delivering drugs or growth factors to specific locations within the body. This capability holds the potential to improve treatment efficacy and minimize side effects.

**Figure 9:**
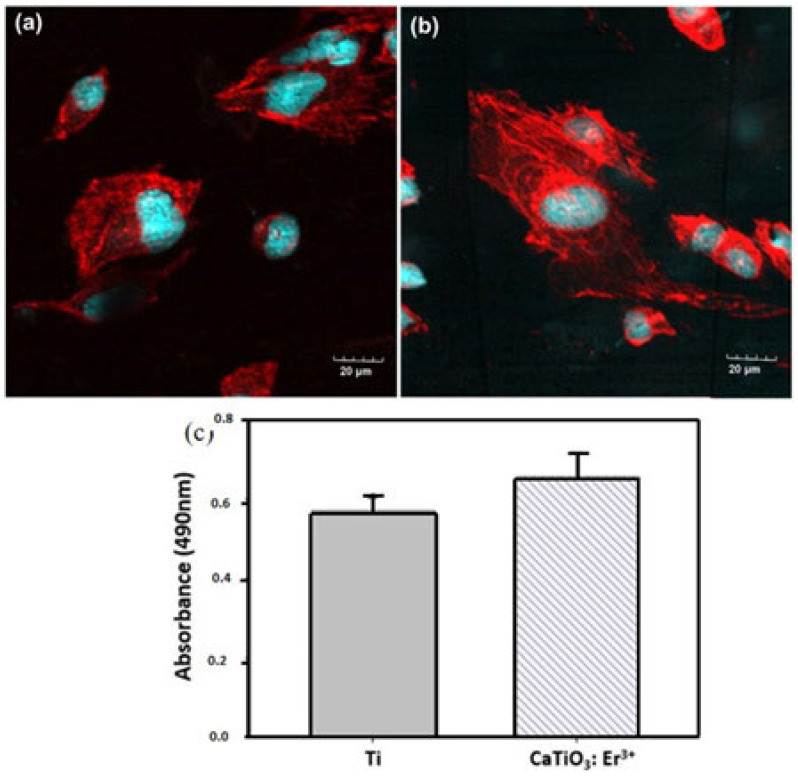
CLMS images of the BHK cell on (**a**) Ti; (**b**) CaTiO_3_: Er^3+^ films; and (**c**) proliferation of the BHK cell on Ti and CaTiO_3_: Er^3+^ films after 72 h of culturing. The red indicates the cytoskeleton structure of the cells and the green indicates the cell nuclei.

To confirm the crucial role of hydrophilicity in biomedical applications, such as enhancing cell adhesion and growth, cell culture experiments were conducted on the material’s surface to evaluate cell proliferation. Confocal laser scanning microscopy (CLSM) images in Figure 9a,b depict BHK cells adhering to the surfaces of Ti and CaTiO_3_: Er^3+^ films, respectively. The uniform distribution and spread-out, fibrous morphology of the cells in both samples indicate good biocompatibility.

An MTS assay was employed to further assess the biocompatibility of the materials with the cell proliferation of the Ti and CaTiO_3_: Er^3+^ films. The rate of proliferation was measured after culturing up to 72 h, using MTS for mitochondrial reduction. This assay is based on the ability of metabolically active cells to reduce a tetrazolium-based compound, MTS, to a purple formazan product. The quantity of formazan product is directly proportional to the number of living cells in the culture. This assay is a widely recognized tool for evaluating a material’s suitability for biological applications [33]. Figure 9c shows cell proliferation on the Ti and the CaTiO_3_: Er^3+^ films. The rate of cell proliferation on the CaTiO_3_: Er^3+^ films was significantly higher than that of the Ti substrate. This suggests that the CaTiO_3_: Er^3+^ films supported the proliferation and growth of BHK cells without inducing any cytotoxic effects. 

## 4. Conclusions

CaTiO_3_: Er^3+^ films were successfully synthesized by a hydrothermal process on a Ti plate. The CaTiO_3_: Er^3+^ films showed a cubic structure, with a bar length of ~5 μm and a width of ~(1–2) μm. The CaTiO_3_: Er^3+^ films displayed highly intensive UC emission under 980 nm laser beam excitation. The emitted light was primarily composed of green and red bands originating from Er^3+^ ions, with maxima at 543, 661, and 740 nm. The CaTiO_3_: Er^3+^ film had excellent surface hydrophilicity, with an almost zero contact angle, which can help increase the application of guide water and good substances in biomedicine. Furthermore, the CaTiO_3_: Er^3+^ film significantly promoted the adhesion and growth of cells. Hence, CaTiO_3_: Er^3+^ films are promising materials for future directions in luminescence and biomedical fields. 

## Data Availability

Data are contained within the article.
